# Palmitoylethanolamide in the Treatment of Chronic Pain: A Systematic Review and Meta-Analysis of Double-Blind Randomized Controlled Trials

**DOI:** 10.3390/nu15061350

**Published:** 2023-03-10

**Authors:** Kordula Lang-Illievich, Christoph Klivinyi, Christian Lasser, Connor T. A. Brenna, Istvan S. Szilagyi, Helmar Bornemann-Cimenti

**Affiliations:** 1 Department of Anaesthesiology and Intensive Care Medicine, Medical University of Graz, 8036 Graz, Austria; 2 Department of Anesthesiology & Pain Medicine, University of Toronto, Toronto, ON M5S 1A1, Canada; 3 Division of Medical Psychology, Psychosomatics and Psychotherapeutic Medicine, Department of Psychiatry, Psychosomatics and Psychotherapeutic Medicine, Medical University of Graz, 8036 Graz, Austria

**Keywords:** palmitoylethanolamide, N-(2hydroxyethyl)-palmitamide, Impulsin, Palmidrol, chronic pain, analgesia, quality of life

## Abstract

Chronic pain is a major source of morbidity for which there are limited effective treatments. Palmitoylethanolamide (PEA), a naturally occurring fatty acid amide, has demonstrated utility in the treatment of neuropathic and inflammatory pain. Emerging reports have supported a possible role for its use in the treatment of chronic pain, although this remains controversial. We undertook a systematic review and meta-analysis to examine the efficacy of PEA as an analgesic agent for chronic pain. A systematic literature search was performed, using the databases MEDLINE and Web of Science, to identify double-blind randomized controlled trials comparing PEA to placebo or active comparators in the treatment of chronic pain. All articles were independently screened by two reviewers. The primary outcome was pain intensity scores, for which a meta-analysis was undertaken using a random effects statistical model. Secondary outcomes including quality of life, functional status, and side effects are represented in a narrative synthesis. Our literature search identified 253 unique articles, of which 11 were ultimately included in the narrative synthesis and meta-analysis. Collectively, these articles described a combined sample size of 774 patients. PEA was found to reduce pain scores relative to comparators in a pooled estimate, with a standard mean difference of 1.68 (95% CI 1.05 to 2.31, *p* = 0.00001). Several studies reported additional benefits of PEA for quality of life and functional status, and no major side effects were attributed to PEA in any study. The results of this systematic review and meta-analysis suggest that PEA is an effective and well-tolerated treatment for chronic pain. Further study is warranted to determine the optimal dosing and administration parameters of PEA for analgesic effects in the context of chronic pain.

## 1. Introduction

The field of pain medicine continues to evolve rapidly, with meaningful progress toward the optimization of treatment for patients with both acute and chronic pain. However, options for pharmacological analgesia are limited to a modest number of substances with variable efficacy and unique risk profiles [1,2,3,4,5]. Consequently, chronic pain remains a major source of morbidity affecting millions of people worldwide, and adequate pain control is unfortunately not ubiquitously delivered. There is widespread interest in both the discovery of novel therapeutics and the repurposing of known agents in pursuit of new evidence-based analgesics for chronic pain.

Palmitoylethanolamide (PEA) is a naturally occurring fatty acid amide which was first isolated and described in 1957 as N-(2hydroxyethyl)-palmitamide [6]. PEA was initially extracted from soybean lecithin, egg yolk, and peanut meal and was reported to have anti-inflammatory properties in an animal model [7]. It was later isolated from mammalian tissues and is an endogenous compound in the human body [8]. The first clinical applications of oral PEA formulations were described (using the trade name Impulsin) five decades ago in former Czechoslovakia. In 1974, Masek and colleagues published a sequence of placebo-controlled double-blind trials evaluating the use of PEA as a respiratory infection prophylactic in a population of 1386 volunteers and reported a significant reduction in pain, amount of fever episodes, and incidence of respiratory tract infections [9]. Impulsin was withdrawn from the market several years later for no apparent reason. However, oral PEA formulations continue to be available as over-the-counter dietary supplements to this day.

PEA is considered a member of the “extended endocannabinoid system” because of its bio-similarity and proposed synergy with the endogenous cannabinoid receptor type 1 (CB1) and 2 (CB2) agonist anandamide (also known as N-arachidonoylethanolamine or AEA), although PEA itself has not demonstrated direct affinity to these receptors [10]. It belongs to the so-called “paracannabinoid messengers” [11]. Mechanistic studies have nevertheless supported a potential analgesic effect for PEA, implicating nociceptor-specific ion channels (e.g., transient receptor potential cation channel subfamily V member 1 (TRPV1)) and nuclear transcription factors (e.g., peroxisome proliferator-activated receptor alpha (PPARα)) in its pharmacodynamic profile. Recently, our group published the results of a randomized crossover study demonstrating clinically relevant analgesic properties of PEA. We used a model of thermal pain, thus investigating its effects on peripheral and central nociceptive mechanisms and on pain modulation [12].

The clinical application of PEA has been described in a variety of contexts, such as in the treatment of peripheral neuropathic pain [13] and musculoskeletal pain [14] and in palliative care [15]. A previous meta-analysis of early clinical trials was undertaken in 2017 and suggested that PEA may be clinically useful in the treatment of chronic pain [16]. However, at that time few studies were available (most of which were of low methodological quality), hindering specific therapeutic recommendations. Several larger and more robust clinical trials have emerged since its publication, warranting an updated evaluation of the potential role for PEA in the treatment of chronic pain. The present systematic review and meta-analysis aims to comprehensively assess the effect of PEA on chronic pain intensity in comparison to placebo or active control in adult populations.

## 2. Materials and Methods

The protocol for this systematic review and meta-analysis was registered in advance with PROSPERO (CRD42021262315), following the PRISMA-P guidelines for preferred protocol reporting [17]. The checklist is available as a supplementary file. This review is investigator-initiated and not funded by any external sources.

### 2.1. Literature Search

The literature search was conducted in two databases: MEDLINE and Web of Science. The search strategy was developed through consultation of a previous bibliometric study on pain diagnoses [18], which formulated an extensive list of pain-related literature search terms. These were supplemented with three additional categories of search terms relating to the intervention (e.g., PEA and other names used to describe the compound), population (e.g., human patients), and study methodology (e.g., randomized controlled trials). An overview of the complete search strategy is provided in Table 1. An additional secondary search was undertaken by manually reviewing reference lists of review articles identified in the primary search. Furthermore, Google Scholar was used to find references not included in the two databases.

Clinicaltrials.gov was searched for registered study protocols. The keywords “palmitoylethanolamide” and “pain” resulted in 13 entries, of which 3 were completed. None of them fulfilled our eligibility criteria.

### 2.2. Study Selection

Our criteria for inclusion in this review were: (1) double-blind randomized controlled trials with either placebo or active control comparators, including both pharmacological and non-pharmacological interventions; (2) description of the intervention of PEA therapy in any formulation (including micronization), dosage, and duration, either alone or in combination with other substances/treatments; (3) adult patients with (4) chronic pain; and (5) description of pain intensity (regardless of assessment tool). Chronic pain was defined as any primary or secondary pain persisting for more than 3 months. We considered original articles, short reports, and letters but excluded meeting abstracts or case series. No restrictions were made on the basis of study location or year of publication.

The initial search findings were filtered to remove duplicates, and the remaining reports were imported to the online software platform Rayyan for screening [19]. Title, abstract, and full-text screening were carried out in sequence by two independent reviewers (KLI and CL) to determine each article’s eligibility. Conflicts were resolved by a third independent reviewer (HBC).

### 2.3. Quality and Risk of Bias Assessment

The quality of included studies was evaluated using the Jadad and modified Jadad score [20]. We chose to apply minimum thresholds of Jadad score ≥3 and modified Jadad score ≥5 for inclusion in the meta-analysis. The risk of bias in each included article was further assessed using Cochrane’s updated risk-of-bias tools for randomized trials (RoB-2) [21]. Assessments of quality and risk of bias were carried out in parallel by two independent reviewers (CL, CK).

### 2.4. Outcomes

The primary outcome of our systematic review and meta-analysis was pain intensity after treatment with PEA or control. We included studies assessing pain intensity with any scale that could be described in sufficient detail, including the Visual Analogue Scale (VAS) and Numeric Rating Scale (NRS).

Chronic pain is often accompanied by functional impairment and a reduction in quality of life [22]. These outcomes are closely tied to enjoyment of physical and social activities, as well as depression [23], and are therefore of critical importance to many patients. For this reason, functional status and quality of life after treatment with PEA or control were examined as secondary outcomes in the systematic review. Additionally, side effects attributable to PEA were included as an additional secondary outcome of the systematic review.

### 2.5. Analysis and Data Management

Pain measurements were rescaled to a standard 11-point scale to calculate mean difference and facilitate inter-study comparison. Primary outcomes were pooled using a random effects model, and a meta-analysis was carried out using RevMan Web [24]. In circumstances where a single study reported more than one interventional group (e.g., different doses of PEA with an additional comparator), their outcomes were considered as independent in the meta-analysis. *p* < 0.05 was considered statistically significant.

The dataset prepared for this systematic review and meta-analysis is available from the corresponding author upon reasonable request.

## 3. Results

**Figure 1:**
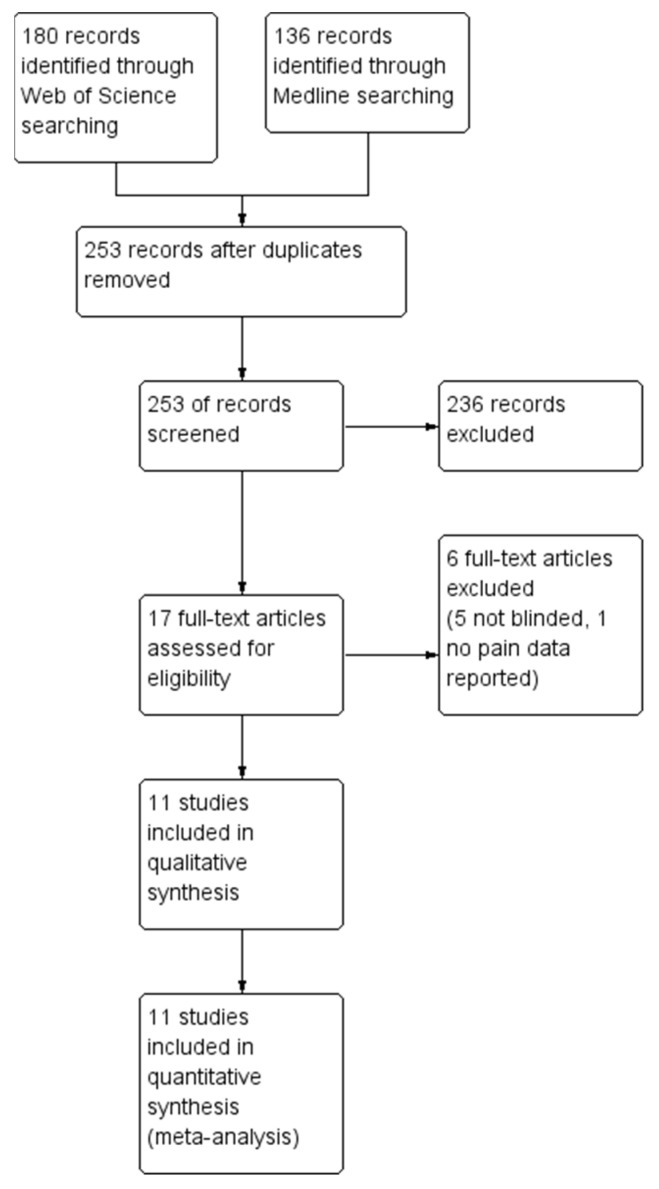
Modified PRISMA flow diagram of study selection.

The final search was carried out on 1 January 2023. A total of 180 articles were identified in the Web of Science database and 136 were identified in MEDLINE. After removal of duplicate reports, 236 were excluded during title/abstract screening and an additional six were removed during full-text screening, leaving 11 articles for inclusion in the narrative synthesis and meta-analysis. A modified PRISMA flow diagram of this process is provided in Figure 1. Included articles ranged in publication date from 2011 to 2022.

### 3.1. Quality and Bias Assessments

**Figure 2:**
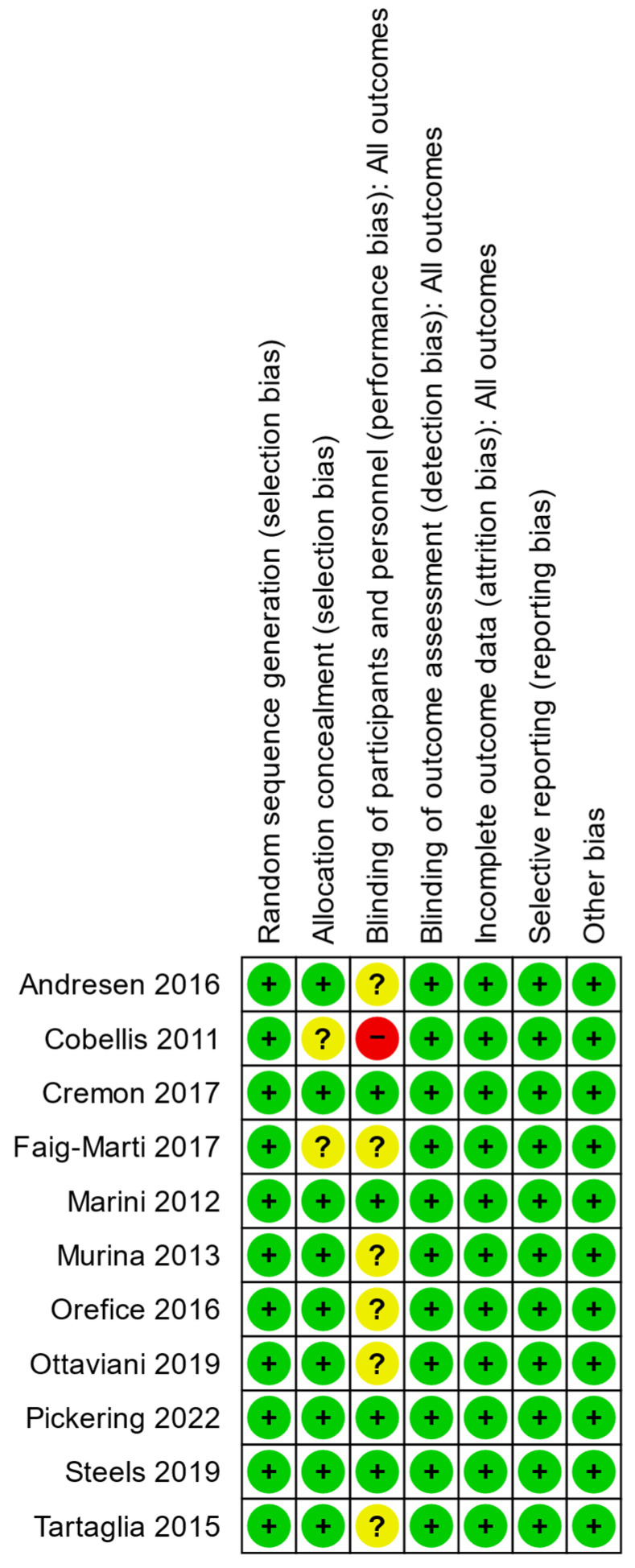
Summary of risk-of-bias assessments for individual articles included in the systematic review. Green +: low risk of bias, yellow ?: unknown risk of bias; red -: high risk of bias.

**Figure 3:**
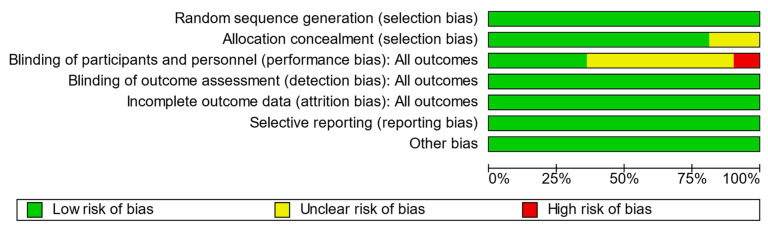
Overview of risk of bias amongst included articles.

Bias assessments for each included study are summarized in Figure 2 and graphed in aggregate in Figure 3. Only one study was scored as high risk of bias in the category for blinding of participants and personnel as it failed to report details on the methods used to achieve blinding. We conducted an auxiliary sensitivity analysis by excluding this study to explore its effect on outcome heterogeneity. Quality assessments of all 11 included studies are presented in Table 2, yielding a mean Jadad score of 3.9 ± 0.8 and a mean modified Jadad score of 6.4 ± 1.1. All studies therefore met the minimum quality requirements of Jadad ≥3 and modified Jadad ≥5 for representation in the meta-analysis.

Two studies were funded by companies [25,26]. In two further studies, the medication was provided by the company [27,28]. None of the studies reported financial disclosures of the authors.

**Table 2:** Summary of quality assessments for individual articles included in the systematic review.

Author, Year	Was the Study Described as Randomized?	Was the Randomization Appropriate?	Was the Study Described as Double-Blind?	Was the Blinding Appropriate?	Were the Dropouts Justified?	Was There a Clear Description of Inclusion and Exclusion Criteria?	Has the Method to Capture Adverse Events Been Described?	Has the Method of Statistical Analysis Been Described?	Jadad Score	Modified Jadad Score
Andresen 2016 [27]	●	●	●	○	●	●	●	●	**4**	**7**
Cobellis 2011 [29]	●	●	●	○	○	●	○	●	**3**	**5**
Cremon 2017 [25]	●	●	●	●	●	●	○	●	**5**	**7**
Faig-Marti 2017 [30]	●	○	●	○	●	●	○	●	**3**	**5**
Marini 2012 [31]	●	●	●	●	○	●	●	●	**4**	**7**
Murina 2013 [32]	●	●	●	○	●	●	○	●	**4**	**6**
Orefice 2016 [28]	●	●	●	○	○	●	●	●	**3**	**6**
Ottaviani 2019 [33]	●	●	●	○	●	●	○	●	**4**	**6**
Pickering 2022 [34]	●	●	●	●	●	●	●	●	**5**	**8**
Steels 2019 [26]	●	●	●	●	●	●	●	●	**5**	**8**
Tartaglia 2017 [35]	●	○	○	○	●	●	○	●	**3**	**5**

Legend: ● = yes, ○ = no.

### 3.2. Study Characteristics and Outcomes

The 11 included studies described a total of 774 patients (383 PEA, 391 control). A majority (70.2%) of patients were female, with individual study percentages ranging from 33.3 to 100%, skewed by three studies which were specific to chronic gynecological pain [29,32,35]. Other treatment indications were pain in neurological diseases [27,28,32,33,34], musculoskeletal disorders [26,31], and irritable bowel syndrome [25]. Study characteristics and extracted data are presented in Table 3 and Table 4.

Seven studies (63.6%) originated from Italy [25,28,29,31,32,33,35], two (18.2%) originated from Australia [26,34], one (9.1%) originated from Denmark [27], and one (9.1%) originated from Spain [30]. Pain intensity was measured using a VAS score in six studies (54.5%), an NRS score in four studies (36.4%), and a Likert scale in one study (9.1%).

All included studies described the administration of oral PEA, with dosages ranging from 300 mg [26] to 1200 mg per day [27,33]. In nine out of 11 studies, PEA was administered twice daily, and in the other two studies it was administered only once daily. Two studies reported on a relatively short duration of treatment (10 and 14 days, respectively) [31,35], while one reported on a 12-month treatment period [28]. The remainder of the included studies applied relatively similar treatment durations of 8 to 12 weeks.

**Figure 4:**
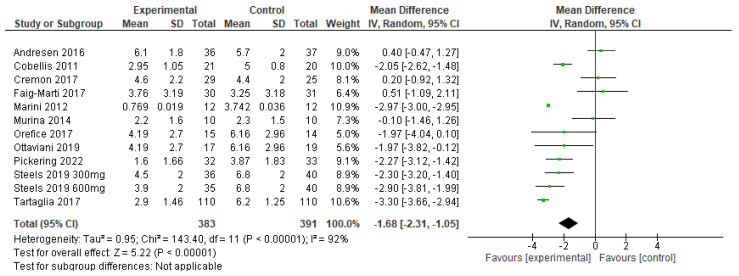
Forest plot of the effect of palmitoylethanolamide (PEA) on chronic pain intensity [25,26,27,28,29,30,31,32,33,34,35]. Experimental refers to PEA, while control refers to placebo or active treatment comparator. Lines represent 95% confidence intervals for each study’s effect size, and the black diamond represents a pooled estimate using a random effects statistical model.

The primary outcome of pain intensity was pooled using a random effects model, and a Forest plot of this meta-analysis is presented in Figure 4. The pooled analysis favored PEA over control treatment, with an average pain intensity reduction of 1.68 (1.05–2.31, *p* = 0.00001) points on a standardized 11-point scale. The effect size favoring PEA was statistically significant (Z = 2.91, *p* = 0.004), although heterogeneity among included studies was high (I2 = 93%).

In addition to analgesic benefit, six of the included studies reported an association between PEA treatment and patient-reported outcome measures, e.g., sleep, quality of life and well-being [25,28,34], or functional status [26,29,31]. One study reported increased general well-being over time in both study groups without significant difference between the groups [25]. Six studies reported no treatment-associated side effects [26,28,29,31,33,34], three studies described no difference in the side effect profile between PEA and placebo [25,27,35], and one study reported a very low incidence of mild and transient gastrointestinal symptoms with PEA [32].

## 4. Discussion

### 4.1. Primary and Secondary Outcomes

Of the 11 studies included in this systematic review, all but two reported significant analgesic benefit for patients treated with PEA. Our meta-analysis of pooled effects favors PEA treatment over control for the treatment of chronic pain, with a mean improvement of 1.68 on an 11-point pain intensity scale.

Several studies also reported an association between improved pain control and commensurate improvement in functional status and quality of life, and no study significantly favored the comparator over PEA for these secondary outcomes. PEA was shown to increase quality of life [28] and improve sleep quality [34] while reducing symptom severity [29] and increasing physical function [26,31]. The latter is of special interest, as both studies which reported a gain in function were conducted in patients with musculoskeletal disorders, i.e., temporomandibular joint arthritis and knee osteoarthritis. These findings are in line with a recent preclinical study which showed an ability of PEA to modify molecular inflammatory mechanisms in a rat model of osteoarthritis [36].

The included studies were heterogeneous with respect to reported side effects. However, only one study suggested unique side effects of PEA compared to a comparator treatment and were deemed mild [32]. This is consistent with the large-scale study carried out by Masek and colleagues in 1974 [9] and with a pooled analysis of nearly 1500 patients which reported no notable side effects associated with PEA therapy [37]. Our findings therefore support the consideration of PEA for patients in whom common analgesics are poorly tolerated due to side effect profile or are otherwise contraindicated.

While the overall findings of our meta-analysis support the application of PEA in the management of chronic pain, we observed a high degree of heterogeneity in the included studies. In particular, studies reported significant methodological variability with respect to: indications for PEA treatment; PEA regimen, including dosage, frequency of administration, and treatment duration; and micronization of the active agent. We explore each of these factors further in the narrative synthesis.

### 4.2. Indication

The included studies describe PEA treatment in the context of a broad spectrum of chronic pain entities. The high degree of heterogeneity in treatment indications presents an obstacle to expressing detailed recommendations in guidelines for the use of PEA to treat specific pain disorders. However, recent meta-analyses have provided evidence for the efficacy of PEA in the treatment of inflammation and neuropathic pain [14,38]. Data from healthy volunteers has also shown that PEA is capable of reducing central sensitization and moderating pain modulation [12], which are desirable features for chronic pain treatments and are consistent with observations from our included studies.

### 4.3. Dosage and Timing

The dosage of PEA varied dramatically between included studies, ranging from 300 mg/day to 1200 mg/day. In six papers, the dose selection was justified by the fact that an equivalent or larger dose had previously been used in other studies [25,26,27,28,33,34]. In five papers, no reasoning for the chosen dosing regime was provided [29,30,31,32,35]. In our dataset, we did not detect a clear dose–effect relationship. This is in agreement with the trial reported by Steels and colleagues in which PEA afforded a significant reduction in pain without any significant difference between patients randomized to either 300 mg/day or 600 mg/day doses [26].

In addition, included reports described both once-daily and twice-daily dosing of PEA. Recent studies of PEA pharmacokinetics have reported that micronized PEA reaches a peak plasma concentration within approximately two hours and falls to levels only slightly above endogenous concentrations after four hours [39]. This may justify future investigations evaluating more frequent dosing regiments (e.g., with meals or four times per day). Nevertheless, significant reductions in chronic pain intensity were reported in studies using either once-daily or twice-daily dosing [28,35].

### 4.4. Duration of Treatment

The duration of treatment was 8–12 weeks among most of the included studies, although outliers included 10-day, 14-day, and 12-month courses of treatment. No clear scientific recommendations exist for the duration of PEA use. Manufacturer recommendations differ depending on indication for treatment.

In the studies by Tartaglia et al. and Marini et al., a short intake period of 10 or 14 days was sufficient to achieve a significant reduction in pain [31,35]. However, most other included studies did not report any significant benefit at comparable early timepoints. Ottaviani et al., Orefici et al., and Steels et al. described reductions in pain and functional impairment after approximately four weeks [26,28,33]. While an optimal duration of treatment has yet to be elucidated for PEA as a chronic pain therapy, on the basis of these studies we suggest that a course of at least four weeks should be considered for pragmatic trials until a robust dose study is undertaken.

### 4.5. Micronization

As a lipophilic compound, the poor water solubility of PEA limits absorption and bioavailability during oral intake [40]. Animal studies and in vitro experiments have already demonstrated that the particle size of PEA plays an important role in the absorption capacity [39,41], providing pre-clinical evidence in favor of treatment with reduced particle sizes or micronization. However, to what extent (if at all) micronization improves the clinical effect is disputed [42].

Of the eleven papers included in our systematic review, three described ultramicronized PEA [27,28,33], two described micronized PEA [29,31], one described PEA co-micronized with an excipient carrier [25], and two described unmicronized PEA [26,34]. In the remaining three articles [30,32,35], the degree of micronization was not explicitly stated and could not be obtained through enquiry to the study authors. One study administering ultramicronized PEA [27] and another administering PEA with an unknown degree of micronization [30] reported no significant difference in pain scores versus comparators. Thus, the importance of particle size for PEA’s analgesic effect is unclear.

The trials reported by Steels et al. and Pickering et al. argue against the need for micronization or ultramicronization of the active ingredient, reporting a clear and significant reduction in chronic pain intensity among patients with knee osteoarthritis using non-micronized PEA compared to placebo [26,34]. While micronized and ultramicronized PEA have shown promising results in animal models and in vitro studies, further pharmacokinetic studies would be required to demonstrate the benefit or necessity of PEA micronization for humans [40].

### 4.6. Strengths and Limitations

In 2017, Artukoglu and colleagues published the first meta-analysis on the efficacy of PEA for pain treatment [16]. Their report was thorough by the standards of the available literature, and they were able to draw the conclusion that PEA was of potential utility as an analgesic. However, a detailed analysis was impeded by highly heterogeneous randomized controlled trials with significant methodological limitations and relatively low quality, as assessed by the authors. Building on their experience, we opted to include only double-blinded randomized controlled trials in our meta-analysis of PEA for chronic pain. As a result, the 11 studies included in our present systematic review performed generally well on assessments of quality and risk of bias, and all studies met our thresholds for inclusion in the meta-analysis. The present study therefore represents a comparatively high-validity report on the use of PEA in chronic pain.

This study also has several limitations. Foremost, although we have searched two major medical databases and performed manual search of reference lists, we may still have missed some trials. However, this limitation is true for every systematic review. Our meta-analysis is limited, however, by highly heterogeneous PEA dosages, dosing intervals, and treatment courses, which may not be fully compensated for by a random effects statistical model. A relatively small number of publications met our strict criteria for inclusion and all but one represented small (<100 patient) trials, potentially limiting statistical power of the pooled estimate. Finally, while our primary study outcome of improvement in pain intensity score does illustrate a statistically significant benefit of PEA on chronic pain scores, this numerical outcome may not necessarily translate into a clinically significant benefit for patients. However, studies in other settings have reported that a 35% reduction in pain scores corresponds to a significant improvement for patients with moderate pain, supporting the notion that PEA may be a valuable adjunct treatment for patients with incompletely managed pain [43].

## 5. Conclusions

Our meta-analysis of double-blind randomized controlled trials reports a pooled effect favoring PEA over placebo or active comparators in the analgesic treatment of chronic pain. PEA was associated with improved functional status and quality of life in many studies, while reported side effects were essentially negligible. While our study advances the possibility of a role for PEA in clinical analgesia, it identifies several important questions that remain unanswered. Future directions for research include elucidating the ideal treatment indications and dosing regimens and further evaluating the relevance of microniziation in head-to-head studies for PEA treatment.

## Figures and Tables

**Table 1:** Overview of the literature search strategy.

Objective	Search Terms
Substance	PEA OR palmitoylethanolamide OR n palmitoyl ethanol amine OR um-pea OR palmidrol OR Impulsin
Population	human OR female OR male OR proband OR patient OR volunteer
Indication	pain OR chronic pain OR acute pain OR neuropathic pain OR nociceptive pain OR allodynia OR analgesia OR arthralgia OR brachialgia OR causalgia OR cephalalgia OR cephalic OR cervicodynia OR colic OR eudynia OR fibromyalgia OR headache OR hyperalgesia OR hypoalgesia OR hyperpathia OR maldynia OR migraine OR neuralgia OR nociceptive OR odontalgia OR opthalmodynia OR vulvodynia OR otalgia OR radiculopathy OR toothache OR orchidodynia OR coccygodynia OR CRPS OR nuchalgia OR lumbalgia OR lumboischialgia OR cervicobrachialgia
Study type	prospective OR randomised OR randomized OR controlled OR observational OR trial

**Table 3:** Characteristics of included studies [25,26,27,28,29,30,31,32,33,34,35].

Author Year	Country	Population	N	Females %	Drop Outs	Dose PEA	Micronization	Manufacturer	Evaluated Pain Scale	COI
Andresen 2016 [27]	Denmark	Spinal cord injury	73	35.2	5/73 (6.8%)	600 mg 2×/d	um	Epitech Group SpA	NRS	Medication provided by Epitech
Cobellis 2011 [29]	Italy	Chronic pelvic pain	61	100	0/61 (0%)	400 mg 2×/d	m	n.r.	VAS	n.r.
Cremon 2017 [25]	Italy	Irritable bowel syndrome	54	50.0	0/54 (0%)	200 mg 2×/d	co-m	Epitech Group SpA	Likert scale	Funded by Company
Faig-Marti 2017 [30]	Spain	Carpal tunnel syndrome	68	60.7	7/68 (10.3%)	300 mg 2×/d	n.a.	Valpharma SpA	VAS	no
Marini 2012 [31]	Italy	Temporomandibular joint arthritis	24	33.3	n.a.	(300 mg + 600 mg)/d (1–7.d), (2× 300 mg)/d (8–14.d)	m	Epitech Group SpA	VAS	n.r.
Murina 2013 [32]	Italy	Vestibulodynia	20	100	0/20 (0%)	400 mg 2×/d	n.r.	n.r.	VAS	n.r.
Orefice 2016 [28]	Italy	Multiple sclerosis	29	51.7	n.a.	600 mg 1×/d	um	Epitech Group SpA	VAS	Medication provided by Epitech.
Ottaviani 2019 [33]	Italy	Burning mouth syndrome	35	82.9	6/35 (17.1%)	600 mg 2×/d	um	Epitech Group SpA	NRS	no
Pickering 2022 [34]	Australia	Diabetic neuropathic pain	70	44.3	4/70 (5.7%)	300 mg 2×/d	no	Gencor Pacific	NRS	no
Steels 2019 [26]	Australia	Knee osteoarthritis	111	53.2	11/111 (12.2%)	150 mg/300 mg 2×/d	no	Gencor Pacific	NRS	Funded by company
Tartaglia 2015 [35]	Italy	Dysmenorrhea	220	100	0/220 (0%)	400 mg 1×/d	n.r.	n.r.	VAS	no

COI: conflict of interest; co-m: co-micronized; m: micronized; n.r.: not reported; NRS: Numeric Rating Scale; PEA: palmitoylethanolamide; um: ultramicronized; VAS: Visual Analogue Scale.

**Table 4:** Key outcomes of included studies [25,26,27,28,29,30,31,32,33,34,35].

Author Year	Intervention	Control	Application	Dose PEA	Duration	Primary Outcome	Secondary Outcome	AE
Andresen 2016 [27]	PEA	Plc.	s.l.	600 mg 2×/d	12 w	No sig. differences	Rescue medication intake in PEA group sig. reduced, no sig. improvement in QoL	no
Cobellis 2011 [29]	PEA + transpolydatin	Plc. or Celecoxib 200 mg 2×/d	p.o.	400 mg 2×/d	3 m	Sig. better pain reduction compared to placebo	Satisfaction with therapy in celecoxib and PEA group sig. higher than in placebo group	no
Cremon 2017 [25]	PEA + transpolydatin	Plc.	p.o.	200 mg 2×/d	12 w	Sig. better pain reduction compared to placebo	General wellbeing questionnaire in both groups without sig. difference Improved, rescue medication intake without sig. differences	no
Faig-Marti 2017 [30]	PEA	Plc.	p.o.	300 mg 2×/d	60 d	No sig. differences	no sig. differences in the two groups in function and seveity	n.r.
Marini 2012 [31]	PEA	Ibuprofen 600 mg 3×/d	p.o.	(300 mg + 600 mg)/d (1–7 d), (2× 300 mg)/d (8–14 d)	14 d	Sig. pain reduction compared to ibuprofen	Change in maximum mouth opening after therapy in PEA group sig. higher than in ibuprofen group	no
Murina 2013 [32]	PEA + transpolydatin	Plc.	p.o.	400 mg 2×/d	60 d	Sig. pain reduction in both groups; no sig. benefit between placebo and PEA	Marinoff Dyspareuniae scale in both groups sig. improves but no sig. difference between placebo and PEA	2 AE’s in PEA, 1 in Plc. Group (mild, transient gastrointestinal symptoms)
Orefice 2016 [28]	PEA	Plc.	p.o.	600 mg 1×/d	12 m	Sig. better pain reduction compared to placebo	QoL with sig. improvement at 12 months compared to placebo, no sig. changes in the EDSS score	no
Ottaviani 2019 [33]	PEA	Plc.	s.l.	600 mg 2×/d	60 d	Sig. better pain reduction compared to placebo	None	no
Pickering 2022 [34]	PEA	Plc.	p.o.	300 mg 2×/d	8 w	Sig. improvement in neuropathic pain scale	Improved sleep quality and mood	no
Steels 2019 [26]	PEA	Plc.	p.o.	150 mg/300 mg 2×/d	8 w	Sig. better pain reduction compared to placebo	WOMAC scores in PEA group sign. better, reduction of rescue medication, improvement in anxiety score, remaining scores unchanged	no
Tartaglia 2015 [35]	PEA + transpolydatin	Plc.	p.o.	400 mg 1×/d	10 d	Sig. better pain reduction compared to placebo	None	no

AE: adverse events; EDDS: expanded disability status scale; n.r.: not reported; PEA: palmitoylethanolamide; Plc: placebo; QoL: quality of life.

## Data Availability

No new data were created or analyzed in this study. Data sharing is not applicable to this article.
